# Limited Capacity for Faster Digestion in Larval Coral Reef Fish at an Elevated Temperature

**DOI:** 10.1371/journal.pone.0155360

**Published:** 2016-05-18

**Authors:** Ian M. McLeod, Timothy D. Clark

**Affiliations:** 1 Centre for Tropical Water and Aquatic Ecosystem Research (TropWATER), College of Science and Engineering and ARC Centre of Excellence for Coral Reef Studies, James Cook University, Townsville, Queensland, Australia; 2 Australian Institute of Marine Science and AIMS@JCU, Townsville, Queensland, Australia; Department of Agriculture and Water Resources, AUSTRALIA

## Abstract

The prevalence of extreme, short-term temperature spikes in coastal regions during summer months is predicted to increase with ongoing climate change. In tropical systems, these changes are predicted to increase the metabolic demand of coral reef fish larvae while also altering the plankton communities upon which the larvae feed during their pelagic phase. The consequences of these predictions remain speculative in the absence of empirical data on the interactive effects of warm temperatures on the metabolism, postprandial processes and growth responses of coral reef fish larvae. Here, we tested the effect of increased temperature on the metabolism, postprandial performance and fine-scale growth patterns of a coral reef fish (*Amphiprion percula*) in the latter half of its ~11-d larval phase. First, we measured the length and weight of fed versus fasted larvae (N = 340; mean body mass 4.1±0.05 mg) across fine temporal scales at a typical current summer temperature (28.5°C) and a temperature that is likely be encountered during warm summer periods later this century (31.5°C). Second, we measured routine metabolic rate (*Mo*_*2* routine_) and the energetics of the postprandial processes (i.e., digestion, absorption and assimilation of a meal; termed specific dynamic action (SDA)) at both temperatures. Larvae fed voraciously when provided with food for a 12-hour period and displayed a temperature-independent increase in mass of 40.1% (28.5°C) and 42.6% (31.5°C), which was largely associated with the mass of prey in the gut. A subsequent 12-h fasting period revealed that the larvae had grown 21.2±4.8% (28.5°C) and 22.8±8.8% (31.5°C) in mass and 10.3±2.0% (28.5°C) and 7.8±2.6% (31.5°C) in length compared with pre-feeding values (no significant temperature effect). *Mo*_*2* routine_ was 55±16% higher at 31.5°C and peak *Mo*_*2*_ during the postprandial period was 28±11% higher at 31.5°C, yet elevated temperature had no significant effect on SDA (0.51±0.06 J at 28.5°C vs. 0.53±0.07 J at 31.5°C), SDA duration (6.0±0.6 h vs. 6.5±0.5 h), or the percent of total meal energy used for SDA (SDA coefficient: 10.1±1.3% vs. 13.0±1.7%). Our findings of higher *Mo*_*2* routine_ but similar SDA coefficient at high temperature provide the first empirical evidence that coral reef fish larvae may have to secure more food to attain similar growth rates during warm summer periods, and perhaps with chronically warmer conditions associated with climate change.

## Introduction

Climate change models predict an increase in sea surface temperatures of up to 4°C this century [1] and an alteration in the plankton communities that form the basis of many food webs [2–4]. Concomitant with these progressive changes, extreme climatic events like summer heat waves are predicted to become more frequent and increase in magnitude as a consequence of global climate change [1]. Indeed, recent evidence has shown that the frequency of anomalously high seawater temperatures has increased along 30% of the world’s coastlines [5]. This is important because there is growing recognition that ecological change is often driven by discrete events in addition to longer-term change [6–8].

The literature on the thermal biology of marine fishes is extensive and has been growing rapidly with the increased focus on climate change research [6,9]. Generally, temperature influences the speed of biochemical reactions and metabolic rates of fishes [10–11], which can have flow-on effects for community and ecosystem functioning by affecting the energy available for growth, foraging and reproduction. Despite this knowledge, the ecological effects of anomalously high temperatures are poorly understood in most marine systems [12–13], perhaps with the exception of coral reefs, where warming of only 1–2°C above the long-term average can cause widespread coral bleaching and mortality [14,15]. The effects of temperature anomalies on the fishes inhabiting coral reefs are less well understood, and are likely to be especially important during the pelagic larval stage.

For many marine fishes, the small pelagic larval stage is considered to be the most vulnerable to environmental stressors such as temperature because key systems (e.g., respiratory and circulatory) are still developing [16,17]. Fish larvae are the smallest vertebrates and can maintain impressive growth rates of up to 100% of body mass per day [18], dependent on temperature, metabolism, food supply/quality and energy-processing efficiency [17,19]. Mortality can be extremely high (i.e., >99%) during the larval stage of many fish species, thus even small changes in mortality rates can have significant impacts on adult populations [19–21]. Larval fishes typically exhibit increased growth rates and shorter pelagic larval durations (PLDs) with increasing temperatures within their natural temperature ranges [22–24], with tropical fish larvae reported to show the most rapid growth and shortest PLDs [25]. Based on this knowledge, it has been predicted that warming waters could have positive outcomes for coral reef fish larvae because enhanced growth will lead to faster metamorphosis and less time in the dangerous pelagic environment [25, 26]. However, recent research has shown that the growth rates of larval coral reef fishes slow during warm summers [27] and in naturally warm equatorial waters [28]. Further, recent research has cast doubt about the hypothesis of faster larval growth in wamer waters once the forecasted interactions between temperature and food availability are considered [29].

Like all vertebrates, larval fishes must expend energy to digest, absorb and assimilate the nutrients from each meal. Termed specific dynamic action (SDA), the energy used during the postprandial processes can contribute significantly to daily energy budgets of most animals [30, 31]. Since the SDA response is dependent on environmental temperature in ectothermic vertebrates [32–33], anomalous warming events have the potential to significantly modify the rate at which meals can be processed and assimilated to achieve growth, yet this has not been examined in larval coral reef fishes.

Using controlled experimental approaches at current-day and forecasted temperatures, we examined food consumption, postprandial processes and growth of larvae of the coral reef damselfish *Amphiprion percula*. This species deposits eggs into the reef matrix and when larvae emerge ~7 days later they disperse to the pelagic environment to feed and grow before returning to a reef at ~11 days post-hatch (DPH) to metamorphose and settle [34]. In the present study, we quantified growth at fine temporal scales by measuring the length and weight of fed and unfed larvae (8 DPH) at a current-day summer temperature (28.5°C) and a forecasted summer temperature (31.5°C) every 3 h for 24 h. Subsequently, to investigate the underlying metabolic physiology associated with the growth data, we determined the routine metabolic rate (*Mo*_*2* routine_) and the postprandial metabolic response (SDA) of larvae (6–9 DPH) at 28.5°C and 31.5°C. Through these measurements, we tested the hypothesis that temperature anomalies will alter the rate of postprandial processes and negatively impact fine-scale growth patterns in coral reef fish larvae.

## Materials and Methods

### Study species and brood stock maintenance

This research was conducted under James Cook University ethics approval A1684. Pairs of the damselfish, *Amphiprion percula*, were captured from the Palm Island region of the central Great Barrier Reef, Australia by Cairns Marine (http://cairnsmarine.com) who have a permit to collect. Fish were transported to James Cook University, Townsville, Australia where they were maintained at the mean temperatures of the source location (22.5–28.5°C annually). Pairs were fed 0.075 g of Aquaculture Nutrition 12/20 pellets (Proaqua Pty Ltd, Queensland, Australia) twice per day and were provided with half a terracotta pot for shelter and to serve as a structure for egg deposition. The pots were inspected each morning during the breeding season for the presence of eggs.

### Larval rearing conditions

On the afternoon when hatching was predicted (6–8 days after egg deposition), pots containing the eggs were transferred to another 60 l aquarium (28.5°C) inside an experimental laboratory, where hatching occurred within a few hours after darkness. Larvae were reared in the 60 l aquarium for two days in a semi-closed system; the system was kept isolated during the day to facilitate feeding, and slowly flushed with filtered seawater each morning prior to light (total water exchange was 2–3 times the volume of the tank). Green *Nannochloropsis* spp. paste (Reed Mariculture, California, USA) was added to the water each morning after flushing until the bottom of the aquarium could not be seen, equating to approximately 4 million cells ml^-1^ [35]. This was done both to dissipate light and maintain the nutritional value of the rotifers (*Brachionus* sp.) that were fed to the larvae at a density of 10 rotifers ml^-1^ each morning for the first two days. From the third day, larvae were fed newly hatched (naupuli) *Artemia sp*. at a rate of two naupuli ml^-1^.

On the morning of the third day post-hatch (DPH), larvae that were visually in good condition (i.e., displaying normal swimming behaviour and balance) were gently collected in a glass beaker and arbitrarily distributed among twelve 3 l culture vessels made of 150 mm polyvinyl chloride pipe as described elsewhere [35]. Between 10 and 20 larvae were stocked in each replicate culture vessel. Three culture vessels were placed in each of four temperature-controlled water baths.

On the fourth DPH, temperatures were increased slowly (1°C every 8 h) over 24 h in half the tanks until the temperature reached 31.5°C. The two temperatures were chosen to represent the current-day summer average in the Palm Island region of the GBR where the brood stock were collected (28.5°C), and a temperature that may be encountered during warm summer periods within this century (31.5°C). The entire protocol was repeated three times using progeny from three adult pairs. The temperature of the water baths and the location of the vessels within the water baths were randomly modified for each run of the experiment to negate any influence of individual vessels or location within the laboratory on results. Larvae raised using this procedure were used for the growth trials and respirometry as described below.

### Growth trials

To establish the rates of growth over 24 h, 8-DPH larval *A*. *percula* were subjected to two feeding regimes (fed and unfed) at the two holding temperatures (28.5°C and 31.5°C) in an orthogonal design. The experimental setup consisted of three 3 l culture vessels per treatment (12 tanks in total). Between 20 and 30 larvae were transferred from the larval rearing vessel to the culture vessels and the treatment temperatures were established as above. Fish were given 12 h to settle prior to the experiment commencing.

Immediately prior to feeding (09:00; time 0 h), 3–10 larvae per tank were killed, blotted dry, weighed (to the nearest 0.1 mg), and photographed in a lateral position on a 0.5 mm plastic grid. The fish in the fed treatments were then fed newly hatched *Artemia* sp. (2 ml^-1^), while the unfed group remained fasted. Subsets of 3–10 larvae per tank were subsequently sampled every 3 h for 24 h. Standard length (SL) to the nearest 0.01 mm was estimated for each fish from the digital photograph using image analysis software (ImageJ version 1.45 s; National Institutes of Health, USA). Larvae weighing less than 2.5 mg (i.e., >1.5 SD below the mean mass of all larvae) were judged to be non-feeding and were discarded from the analyses (N = 24). The entire protocol was run three times using progeny from three pairs of *A*. *percula* to achieve desired sample sizes (340 larvae among treatments).

Condition factor was calculated as the mean residual values after regressing the lengths and weights of all larvae in the growth trials. The condition factors at each temperature are displayed in Table 1. Independent sample t-tests showed there were no significant effects of temperature for either the fed or the unfed fish on larval length, weight, or condition factor at the end of the growth experiment (Table 1). As such, the results were pooled between temperatures to produce an overall fed group and an overall unfed group for all further analyses, although the temperatures are often presented separately for illustrative purposes. Differences in mean weight, length, or condition factor between fed and unfed larvae at each time of sampling were assessed using independent sample t-tests. Differences in length, weight or condition factor for fed and unfed larvae over time were assessed using ANOVA with a post-hoc two-sided Dunnett’s test using time 0 as the control category (Table 2).

**Table 1:** Summary of larval weight (mg), length (mm) and condition factor (means ± SE) for fed and unfed larvae at the end of the growth experiment at 28.5°C and 31.5°C. The *d*.*f*., t and *P* values show results of Independent Sample t-tests with temperature as the grouping variable.

*Variable*	*28*.*5 (*°*C)*	*31*.*5 (*°*C)*	*d*.*f*.	*t*	*P*
Weight (fed)	4.49±0.17	5.01±0.21	19	-4.7	0.64
Weight (unfed)	3.24±0.15	3.33±0.10	19	-1.1	0.92
Length (fed)	5.11±0.07	5.40±0.05	19	0.24	0.24
Length (unfed)	4.98±0.08	4.95±0.46	19	-1.6	0.17
Condition factor (fed)	0.21±0.17	0.16±0.21	19	-0.93	0.37
Condition factor (unfed)	-0.79±0.12	-0.65±0.10	19	-1.1	0.28

**Table 2:** ANOVA outputs comparing larval *Amphiprion percula* weight (mg), length (mm) and condition factor. Differences were identified using Dunnett’s post hoc tests using time 0 (the start of the experiment) as the control category.

Effect	*d*.*f*.	*Mean Square*	*F*	*P*
*28*.*5°C*				
Weight (fed)	8	5.71	13.1	<0.001*
Weight (unfed)	8	0.43	1.29	0.26
Length (fed)	8	0.74	8.89	<0.001*
Length (unfed)	8	0.22	2.88	0.007*
Condition factor (fed)	8	1.50	6.77	<0.001*
Condition factor (unfed)	8	0.43	1.29	0.26
*31*.*5°C*				
Weight (fed)	8	2.85	3.27	0.003*
Weight (unfed)	8	0.37	1.20	0.31
Length (fed)	8	0.59	5.23	<0.001*
Length (unfed)	8	0.07	0.87	0.54
Condition factor (fed)	8	2.52	6.08	<0.001*
Condition factor (unfed)	8	0.44	2.03	0.58
Condition factor (unfed)	8	0.44	2.03	0.58
Combined (*28*.*5°C* + *31*.*5°C*)				
Weight (fed)	8	7.44	11.7	<0.001*
Weight (unfed)	8	0.56	1.77	0.88
Length (fed)	8	0.98	9.21	<0.001*
Length (unfed)	8	0.22	2.80	0.06
Condition factor (fed)	8	3.52	11.2	<0.001*
Condition factor (unfed)	8	1.56	7.87	<0.001*

* Indicates statistically significant difference from control category at time = 0.

### Respirometry

Custom-built respirometers and intermittent-flow respirometry were used to determine postprandial oxygen consumption rates (*Mo*_*2*_) of a subset of 6–9 DPH larval *A*. *percula*. Larvae were gently transferred using a plastic pipette from the larval rearing vessels into individual 4.8 ml glass vials that served as respirometry chambers. Four respirometers were used in each trial and they were submerged in an aquarium maintained at the same temperature at which the larvae were reared. To reduce background microbial respiration, seawater used for the respirometers was UV-sterilized, and the system was cleaned with 70% ethanol before each trial. Background respiration was quantified in all chambers before each trial, and in all cases was negligible. Water was continuously recirculated within each respirometer using a closed circuit of tubing individually connected to a peristaltic pump, which ensured homogeneous oxygen tension throughout the apparatus. The total volume of the respirometer chamber and tubing circuit was 10±1 ml (exact volumes used for *Mo*_*2*_ calculations). A submersible pump connected to a timer was used to flush the respirometer chambers with aerated water (5 ml min^-1^) for 15 min in every 30 min period, and excess water flushed from each respirometer overflowed from a small standpipe that extended just above the water surface in the aquarium. Temperature-compensated oxygen concentration of the water within each chamber was continuously recorded (0.5 Hz) using oxygen-sensitive REDFLASH dye on contactless spots (2 mm) adhered to the inside of each chamber and linked to a Firesting Optical Oxygen Meter via fibre-optic cables (Pyro Science. K., Aachen, Germany). Once all four respirometers contained one individual larvae, the main lights in the experimental room were turned off such that only dim lighting remained, and the trials commenced and ran for 10.5 h. Oxygen consumption rates were calculated from the decline in oxygen concentration over time during the 15 min period between flush cycles. At the end of the respirometry trials larvae were killed, blotted dry with a paper towel and weighed with scales accurate to 0.1 mg. After the larvae were removed from the chambers, three 15 min blanks were run with a 5 min flush cycle between each to quantify any changes in background microbial respiration rates since the beginning of the trial.

Given the large surface area to volume ratio of the respirometers, it was imperative to account for background *Mo*_*2*_ during the trials. To quantify the build-up of background *Mo*_*2*_, 12 blank trials without fish were run at each temperature for 10.5 h. Analysis of these blanks revealed that the build-up of background *Mo*_*2*_ fitted a sigmoidal function. The average *Mo*_*2*_ of the three blank trials at the end of each experiment was used as the end point for further back-calculation. The function of the mean for each temperature at each half-hour time period was used to fit background *Mo*_*2*_ for each fish. Background *Mo*_*2*_ was subtracted from fish *Mo*_*2*_ dynamically across the 10.5 h duration of the trials.

To account for the difference in weight while the postprandial fish grew overnight in the respirometers, we assumed a linear increase in weight between the group sampled at time 0 h (representing the unfed control group; see ‘Growth trials’) and the fed fish sampled at time 24 h (when the gut was empty and postprandial processes complete), and mass-specific *Mo*_*2*_ (i.e., mgO_2_ kg^-1^ h^-1^) was calculated dynamically throughout the trials.

The energy content of *Artemia* sp. in the gut of each larvae (after 12 h of access to food) was estimated by assuming a linear increase in weight of the fish (not including food in the gut) during the 24 h of the experiment, then using the mean weights from the growth experiment to: (1) back-calculate the fish weight at 12 h, (2) back-calculate the weight of the fish plus the *Artemia* in its gut at 12 h, (3) subtract point 1 from point 2 to estimate the weight of *Artemia* in the gut when the individual fish were placed into the respirometry chambers, and (4) calculate meal energy intake using the energy content of newly hatched *Artemia* sp. shown in Table 3.

**Table 3:** Nutritional content of the newly hatched *Artemia sp*. fed to the larval *Amphiprion percula*.

Constituent	(%)
Lipid	2.364
Protein	7.009
Ash	0.860
Moisture	87.07
Nitrogen free extract (digestible carbohydrate)	2.690
Energy (J g^-1^)	2511*

Nutrient content of newly hatched *Artemia sp*. was obtained from David Francis, Australian Institute of Marine Science (unpublished data).

* Energy content calculated assuming that lipid, protein and carbohydrates have energy equivalents of 37.70 KJ g^-1^, 16.77 KJ g^-1^, and 16.77 KJ g^-1^, respectively (German 2011).

Respirometry data for 23 postprandial larvae, 20 unfed larvae, and 24 blank trials without fish were analysed using LabChart version 6.1.3 (ADInstruments, Sydney, Australia). The duration of the postprandial increment in metabolic rate (SDA duration) was calculated as the time between when the larvae were removed from access to food and placed in the respirometer, and the return of *Mo*_*2*_ to stable baseline levels. *Mo*_*2*_ was considered to be at baseline levels and postprandial processes complete when the 1.5-h running mean *Mo*_*2*_ (i.e., three successive *Mo*_*2*_ measurements) was within two standard deviations of the minimum 1.5-h mean *Mo*_*2*_ [36].

Using the baseline *Mo*_*2*_ levels at the end of the postprandial period, specific dynamic action (SDA) was calculated for each individual as the excess energy expended above baseline throughout the postprandial period. Conversion of *Mo*_*2*_ to its energy equivalent was performed assuming 14.32 J of energy was expended per 1 mg of O_2_ consumed [37]. Peak metabolic rate during the SDA process (*Mo*_*2*peak_) was calculated as the highest mean *Mo*_*2*_ over any 1.5 h period (i.e., three successive *Mo*_*2*_ measurements). Factorial scope was calculated for each individual as *Mo*_*2* peak_ / *Mo*_*2* routine_. The SDA coefficient was calculated as the percentage of meal energy that was used during the SDA process. The Q_10_ temperature coefficient for the measured variables was calculated using the formula: Q_10_ = (R2 / R1)^10 / (T2—T1)^ where T1 and T2 are the temperatures over which the change was recorded, R1 is the rate of a process at T1, and R2 is the rate of the same process at T2. Differences in metabolic variables between temperatures were assessed using independent sample t-tests.

### Nutritional composition of Artemia

The nutritional composition of the *Artemia* was determined via proximate composition analysis according to standard procedures. As in [38] moisture content was determined by drying samples in an oven at 80°C to constant weight, protein (Kjeldahl nitrogen; N×6.25) was determined in an automated Kjeltech (Model 2300, Tecator, Höganäs, Sweden), total lipid was measured using chloroform/methanol (2/1 v/v) extraction [39], and ash was measured after incineration in a muffle furnace (Model WIT, C & L Tetlow, Blackburn, Victoria, Australia) at 550°C for 18 h.

## Results

### Growth

**Fig 1:**
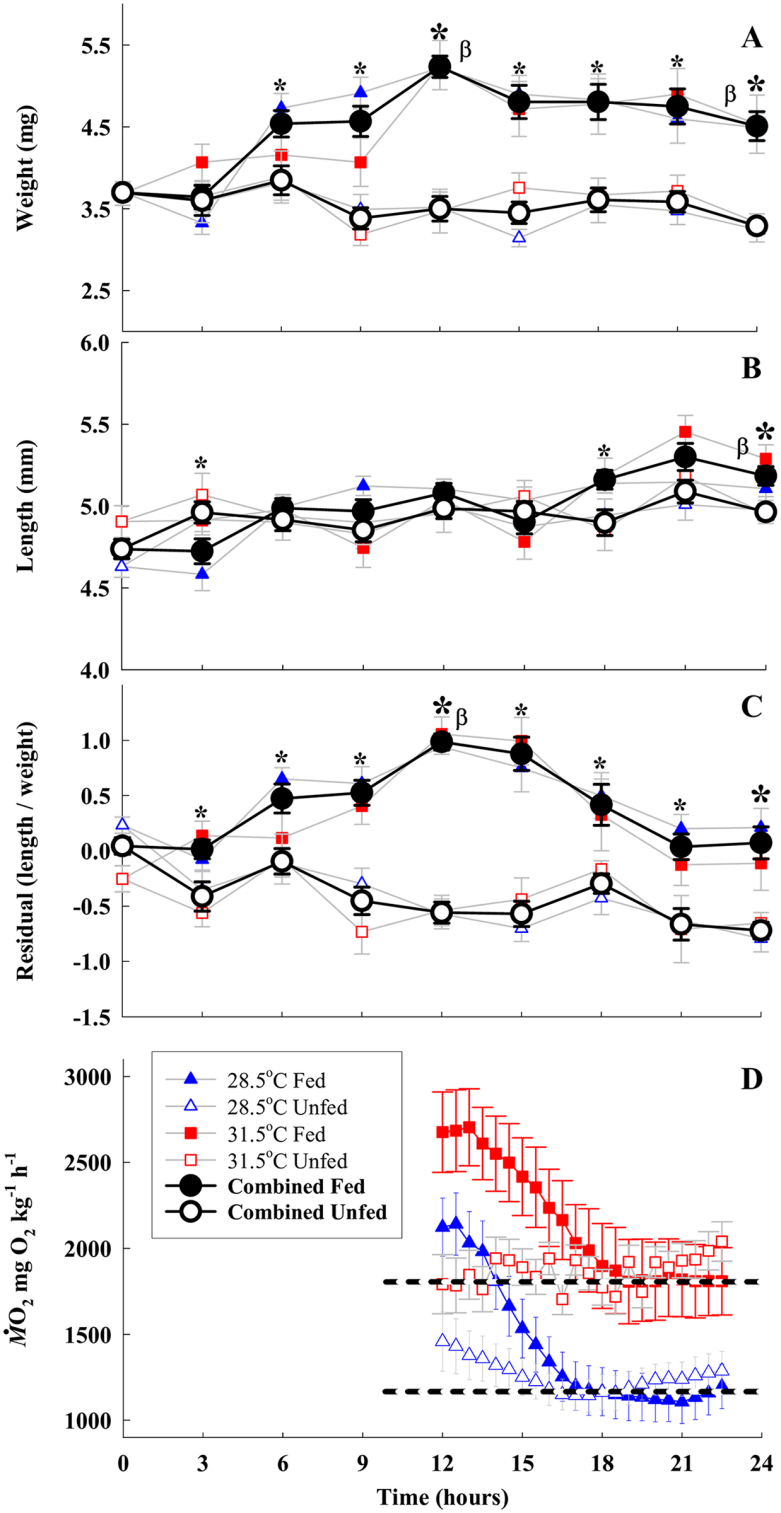
Mean (±SE) weight (A), standard length (B) and condition factor (C), of fed (filled symbols) and unfed (open symbols) larval *Amphiprion percula* at 28.5°C (blue) and 31.5°C (red) over 24 h, and metabolic rate (D) over 12 h. Data for each of the two temperatures are presented as blue and red symbols in the background of (A) through (C), but temperatures were combined (black symbols) after statistical analyses revealed no differences between temperatures (see text). * denotes significant difference between fed and unfed treatments within a time point. β denotes significant differences compared with time 0 at 12 h (beginning of food restriction for fed fish) and 24 h (once postprandial processes were complete). Open symbols in (D) represent unfed fish, while filled symbols represent fed fish. The horizontal black dashed lines in (D) show *Mo*_*2* routine_ at 28.5°C (lower line) and 31.5°C (upper line). Statistical analyses for the data in (D) are presented in the text.

Temperature did not have a significant effect on any of the measured growth parameters, so both temperatures were combined for subsequent analyses (Table 1; although results at each temperature are often given individually, as in Fig 1A–1C).

Larvae in the fed treatments were 41.6±4% (mean±SE) heavier (41.0% at 28.5°C, 42.6% at 31.5°C), 7.2±2% longer (8.5% at 28.5°C, 5.7% at 31.5°C), and had a higher condition factor after 12 h of access to food, reflecting the full gut and initial stages of the growth phase of the fed fish (Fig 1A–1C). After 24 h (12 h access to food then 12 h of fasting) fed larvae were 22.0±5% heavier (21.2% at 28.5°C, 23.0% at 31.5°C) and 9.4±2% longer (10.3% at 28.5°C, 7.8% at 31.5°C) than the control fish that were sampled at the start of the experiment (Fig 1, Table 2). A return of the condition factor to pre-feeding levels (Fig 1C), and visual verification that the gut was empty, indicated that these changes were due to growth. In contrast, unfed fish had no significant change in weight (14% lighter at 28.5°C, 10.7% lighter at 31.5°C), were 4.8±2% longer (7.4% at 28.5°C, 1.0% at 31.5°C), and had a lower condition factor after 24 h (Fig 1A–1C).

### Metabolism

Peak *Mo*_*2*_ during the postprandial process and *Mo*_*2* routine_ at the completion of the postprandial process were 27.8±11% (Q_10_ 2.3; *p*<0.03) and 55.0±16% (Q_10_ 4.0; *p*<0.05) higher at 31.5°C than 28.5°C, respectively. However, the elevated temperature had no significant effect on SDA (0.51±0.06 vs. 0.53±0.07 J at 28.5°C and 31.5°C, respectively; Table 4), SDA duration (5.95±0.61 vs. 6.54±0.46 h, respectively; Table 4), or the percent of total meal energy used during the SDA process (SDA coefficient; 10.14±1.34 vs. 12.96±1.74%, respectively; Table 4). Post-SDA *Mo*_*2*_ did not significantly differ from unfed *Mo*_*2* routine_ at either temperature (28.5°C *p* = 0.17; 31.5°C *p* = 0.40; Fig 1D; Table 2), providing evidence that the postprandial processes were complete.

**Table 4:** Summary of *Mo*_*2*_ and SDA results (means ± SE) for larval *Amphiprion percula* at 28.5°C and 31.5°C. *P* value refers to comparisons between temperatures.

*Variable*	*28*.*5°C*	*31*.*5°C*	*P*	*Q*_*10*_
Unfed *Mo*_*2* routine_ (mg kg^-1^ h^-1^)	1235 ± 89.8	1873 ± 247	0.029*	4.03
Post-SDA *Mo*_*2*_ (mg kg^-1^ h^-1^)	1111 ± 126	1722 ± 175	0.010*	4.24
*Mo*_*2* peak_ (mg kg^-1^ h^-1^)	2208 ± 178	2820 ± 211	0.033*	2.26
SDA duration (h)	5.95 ± 0.61	6.54 ± 0.46	0.218	
Meal energy (J)	5.32 ± 0.29	4.14 ± 0.21	0.003*	
SDA (J)	0.51 ± 0.06	0.53 ± 0.07	0.612	
SDA coefficient (%)	10.14 ± 1.34	12.96 ± 1.74	0.106	

* Indicates significant differences between temperatures.

## Discussion

This study is the first to quantify fine-scale patterns in metabolism and growth of a larval coral reef fish to understand the rate at which a satiation meal is processed and converted into gross mass gain. We found that larval *A*. *percula* were voracious predators, showing extremely distended abdomens and a 41.6±4% increase in body mass during the 12-h feeding period. When access to food was removed and the fed larvae were given 12 h to complete postprandial processes, body length and mass plateaued at values that were, respectively, 9.4±2% and 22.0±5% higher than the pre-feeding control values.

Measurements of the postprandial metabolic response provided some insight into the underlying mechanisms associated with such high growth rates in larval coral reef fishes. While it has been previously documented that larval coral reef fish maintain exceptional rates of routine aerobic metabolism [40], no previous study has investigated the metabolic responses associated with the digestion, absorption and assimilation of a meal. This represents a significant knowledge gap, since postprandial processes are likely to be fundamental components of the daily energy budget of pelagic larval fishes as they strive to attain the critical body size necessary for settlement and metamorphosis.

*Amphiprion percula* larvae had an SDA coefficient of 11.7±1% (combined temperatures) when feeding on *Artemia*, indicating that around 12% of meal energy was expended during digestion, absorption and assimilation. This represents a greater efficiency than the >25% reported for most other fishes when feeding on natural foods [32] but is similar to the growth rate of 13% shown by larval whitefish, *Coregonus lavaretus* when fed *Artemia* [33]. Interestingly, we found no evidence for a reduced SDA duration at 31.5°C in comparison with fish at 28.5°C, which contrasts with the general observation for ectothermic vertebrates that SDA duration decreases with temperature [32]. This may have implications for meal processing capacity in warmer environments (e.g., summer heat waves), as a greater food intake or improved digestive efficiency may be required to satisfy higher baseline metabolism prior to partitioning excess energy into growth. Indeed, the finding of similar SDA duration between the two temperatures suggests that SDA cycles may necessarily overlap under warm conditions if the larvae are to obtain sufficient energy to ensure growth rates are not compromised. Future research examining the plasticity in the efficiency of meal processing and cellular ATP production under chronically warm conditions will shed light on the long-term impacts of climate warming on the energetics and growth potential of larval coral reef fishes.

The peak of the SDA response is thought to be associated with large allocations of energy into protein synthesis [41]. Due to the generally limited metabolic scope in fish larvae [42], any short-term allocation of energy into protein synthesis may limit the energy that can be allocated to other energy-demanding processes. Indeed, reduced activity after feeding has been observed in a number of taxa including adult fishes [43], and has been suggested for larval fishes based on measurements of aerobic metabolism [44,45]. If a warmer environment necessitates more regular and overlapping SDA events as proposed in the present study, larval fishes may be compromised in their capacity to simultaneously perform other important aerobic processes such as sustained swimming.

The dominant prey items for low latitude marine fish larvae are copepods from the Calanoidae and Cyclopidae families and other crustacean larvae (nauplii) [46, 47]. Copepods have a higher nutritional value than *Artemia* [48], yet the relative digestive effort required to process these two food items by larval damselfishes is unknown. Repeating the current experiments using copepods as the food source would provide useful comparisons with the present study. Moreover, it would be valuable to better understand the roles of prey switching and feeding optimisation to the growth of larval coral reef fishes in the wild.

The novel, controlled experiments conducted in the present study give rise to a deeper understanding of how high temperatures may influence larval coral reef fishes. Water temperatures in coastal marine systems can be anomalously warm due to factors such as summer heat waves and influxes of warm currents. A 3°C increase in temperature in the present study caused a 55.0±8% increase in *Mo*_*2* routine_, which would necessitate a significant increase in food consumption just to maintain baseline metabolism if larvae were transiently exposed to these conditions in the natural environment. This may leave less energy available for growth, especially when food supplies are low. McLeod et al. [27] showed that elevated temperatures coupled with restricted food supply dramatically reduced growth rates in larval *A*. *percula*, consequently leading to a significantly longer PLD, which would likely translate to reduced survival. These impacts may be more severe in equatorial regions where waters are naturally warmer, as early evidence shows that the growth rates of some species of coral reef fish larvae are currently suboptimal in equatorial regions [28]. If high temperature anomalies become more frequent and plankton communities concomitantly become more ephemeral [4], our results suggest that the success of coral reef fish larvae may become even more variable and patchy into the future, potentially influencing population dispersal, connectivity and persistence.
